# Leveraging the health equity implementation framework to foster an equity focus in medical education

**DOI:** 10.1007/s10459-023-10277-0

**Published:** 2023-09-05

**Authors:** Deepa Ramadurai, Judy A. Shea

**Affiliations:** 1 Division of Pulmonary, Allergy and Critical Care Medicine, Hospital of the University of Pennsylvania, Philadelphia, PA, USA; 2 Leonard Davis Institute of Health Economics, University of Pennsylvania, Philadelphia, PA, USA; 3 Division of General Internal Medicine, University of Pennsylvania, Philadelphia, PA, USA; 4 Perelman School of Medicine, University of Pennsylvania, Philadelphia, PA, USA

**Keywords:** Health equity, Graduate medical education, Implementation science, Health equity implementation framework

## Abstract

Teaching equitable clinical practice is of critical importance, yet how best to do so remains unknown. Educators utilize implementation science frameworks to disseminate clinical evidence-based practices (EBP). The Health Equity Implementation Framework (HEIF) is one of these frameworks, and it delineates how health equity may be concomitantly assessed and addressed in planning the implementation of an EBP. The HEIF therefore lays a strong foundation to understand and explain barriers and facilitators to implementation through an equity lens, making it well-suited for use by medical educators. Three equity-focused frames of reference within the model include (1) the clinical encounter, (2) societal context, and (3) culturally relevant factors, herein referred to as domains. The HEIF provides a structure for prospective and retrospective assessment of how EBP are taught and ultimately incorporated into clinical practice by trainees, with specific attention to delivering equitable care. We present three examples of common topics in internal medicine, contextualized by the three equity domains of the HEIF. We additionally acknowledge the limitations of this framework as a research tool with complex features that may not be suitable for brief teaching in the clinical environment. We propose a 360-degree learner assessment to ensure implementation of this framework is successful. By encouraging trainees to explore the narrative experiences of their patients and examine their own implicit biases, the HEIF provides a structure to address gaps in knowledge about delivering equitable care.

## Introduction

**Fig. 1:**
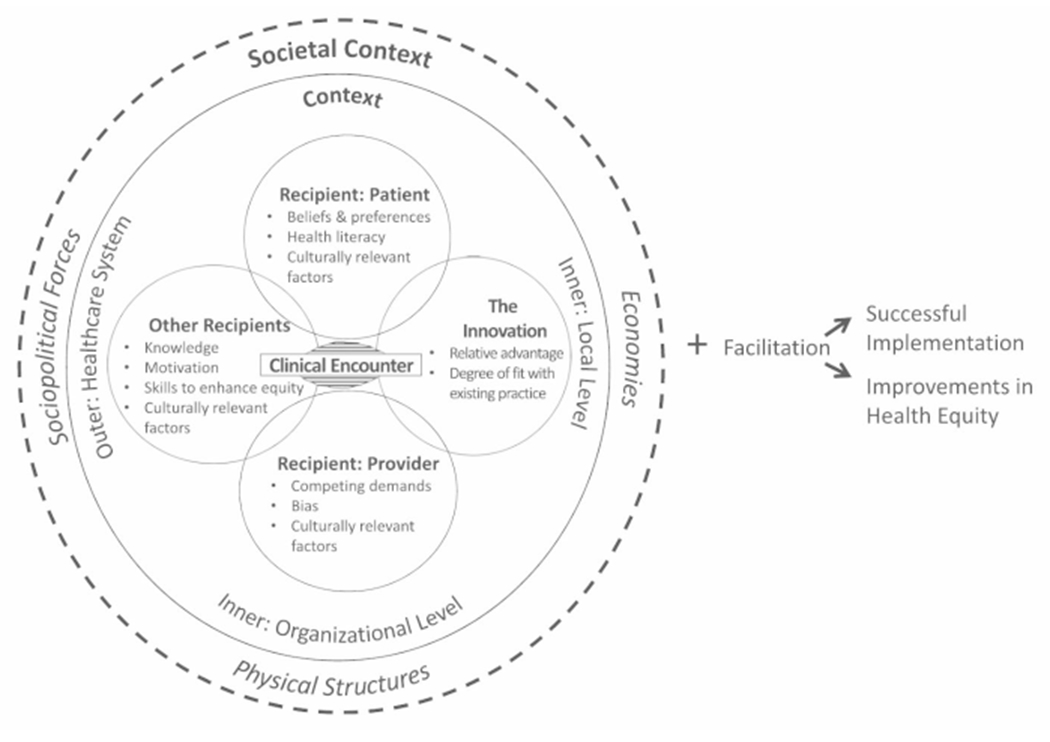
The Health Equity Implementation Framework (Woodward et al., 2021) with pre-specified determinants believed to predict successful and equitable implementation, grouped by domain

Education on inclusivity, diversity, and equity is an international priority in physician training (AAMC, 2020, 2021; ACGME, 2022; Council, 2012; *Transforming and scaling up health professionals’ education and training*, 2013). Despite recommendations to routinely incorporate this education into graduate medical education, a gap remains in providing a structure for how to do so (Fair M, 2021; Maldonado et al., 2014; Smith, 2021). One promising option is the Health Equity Implementation Framework (HEIF) (Woodward, Matthieu, Uchendu, Rogal, & Kirchner, 2019), which is a conceptual framework that promotes the systematic uptake of evidence-based practices (EBPs) into routine practice (Nilsen, 2015). The HEIF (Woodward et al., 2019) (Fig. 1) is a research tool that supports integration of innovative EBPs in patient care (Emmons & Chambers, 2021; Haddad et al., 2020; Leone et al., 2020; Nilsen, 2015). The HEIF also models how educators could instruct trainees on equitable delivery of EBPs, while providing a systematic way to explain barriers and facilitators to EBP implementation both prospectively and retrospectively (Price et al., 2015). Although the primary intent of the HEIF is to provide a structure for incorporating equity principles into the delivery of an EBP, the framework exposes gaps related to teaching about equity within medical education. Beyond the issues with a lack of structure, reasons for why equity principles are not incorporated into medical education might include lack of resources or expertise, time constraints related to curriculum reform, and institutional priorities. The HEIF may not necessarily expose these alternative issues, and it is important to therefore understand its limitations.

Determinant frameworks, such as the HEIF, incorporate features of individuals, organizations, communities, systems, and policies that ultimately determine successful implementation of an EBP (Woodward et al., 2021). Determinant frameworks are organized into domains, which often cross between the aforementioned groups (e.g., individuals, organization, communities, etc.). The central domain of this framework (i.e., where the EBP is discussed and delivered) is the clinical encounter. Recipients, in three overlapping circles, are immediately proximal to the clinical encounter, and all directly influenced by culturally relevant factors. The clinical encounter is lastly connected to the innovation or EBP itself, which is typically a procedure, program, practice, pill, or policy (Woodward et al., 2021). The first encompassing circle is context, which includes inner and outer regions. Context accounts for people, culture, and resources on micro (e.g., local in a clinic), meso (e.g., organizational in a hospital), and macro (e.g., health system) levels. The outermost circle, or societal context, includes economies, physical structures, and sociopolitical forces. These are historically engrained in an environment (e.g., discrimination), and therefore envelop everything within that environment. To the right, facilitation implies the process by which all determinants are addressed, involving all key stakeholders. The ultimate goal is successful EBP implementation and improvement in health equity.

The HEIF merges a determinant framework in implementation science, Integrated-Promoting Action on Research Implementation in Health Services (i-PARIHS) (Harvey & Kitson, 2016), with the Health Care Disparities Framework created by researchers and clinicians from the Veterans Administration (Kilbourne, Switzer, Hyman, Crowley-Matoka, & Fine, 2006). The i-PARIHS framework focuses on the innovation or EBP in the context of local, organizational, and system-level context. i-PARIHS is grounded upon Roger’s Diffusion of Innovations Theory, which highlights how characteristics of knowledge contribute to how the knowledge is perceived, accepted, or incorporated, and Weiner’s theory of organizational readiness to change, which links the behavior of implementation to wanting to change and ability to change. These learning theories in combination create an implementation framework that is dynamic with a practical way for a facilitator to introduce an innovation. In parallel to this framework, the Health Care Disparities Framework delineates three phases of health disparities research (i.e., detection, understanding, and reduction or elimination), with each phase framed according to the clinical encounter, patient factors, provider factors, and health system factors. The theoretical underpinning is to focus on methodological issues in health system-level health disparities, rather than simply describing causes of inequities or improving documentation of inequities. Built from a seminal research article that identified the issues with racial variations in health outcomes (Rathore & Krumholz, 2004) and the Institute of Medicine’s “Unequal Treatment” (Medicine, 2002), the health disparities framework expands the definition of health disparities to include differences in both health outcomes or health status and thereby expands the definition of vulnerable populations beyond race and ethnicity and includes access to care and patient preferences as potentially intervenable individual factors that mediate disparities. Combining i-PARIHS and the Health Care Disparities Framework provides a representation of the stakeholders, multiple levels of environment, and unique components of identifying equity and inequity brought together to form the HEIF.

To meet the needs of integrating equity principles into implementation science frameworks, the HEIF incorporates three broad domains that affect health equity: (1) the clinical encounter, (2) societal context, and (3) culturally relevant factors (Woodward et al., 2021). With these three domains in mind, educators may call out the specific determinants that may implicate how and when EBPs are recommended, and which practices promote equitable care, with an understanding that implicit bias may play a role in any and all domains. We propose to use the HEIF therefore with examples from internal medicine based in both the educator-trainee dyad and the patient-trainee dyad (Table 1). We additionally suggest measurable outcomes to ensure the fidelity of framework implementation.

Alternative implementation science frameworks with purposeful integration of health equity principles exist, however, have different a different audience or context making them less amenable to use in medical education. For example, the Transcreation Framework focuses only on a community setting for implementation (Napoles & Stewart, 2018). The Equity-focused Implementation Research (EquIR) importantly links population health status before and after the intervention, yet maintains much more of a research focus, requiring a specific research question to be employed at the start of the framework with subsequent considerations of social determinants of health based off of the research question alone (Eslava-Schmalbach et al., 2019). Finally, an extension of the RE-AIM (Reach, Effectiveness, Adoption, Implementation, and Maintenance) framework focuses on the evaluation of implementation, and specifically focuses on sustainability of the intervention (Shelton, Chambers, & Glasgow, 2020). It is, however, equally important to recognize and address alternative implementation outcomes including feasibility, acceptability, and fidelity, given the nascent understanding of health equity within EBP discussion in medical education.

### Domain 1. Clinical encounter

The clinical encounter consists of the trainee-patient interaction, where decisions about diagnosis and treatment are made. Educators may desire to focus on helping trainees relate directly to patients’ lived experiences and realizing predetermined perceptions that clinicians may carry into patient interactions. Acknowledging the power dynamic between trainee and patient is also crucial in teaching equity in this setting. The primary feature of this domain is identifying and addressing non-verbal behaviors that may be perceived as dismissive or verbal cues that indicate lack of concern for the patient. This could include interruptions, disregard of concerns, smiling or lack thereof, and eye contact amongst others. Simulation, videotaping, and prompted reflection could emphasize how biases are identified and addressed, how trust is developed, and how recommendations are framed.

When applying this domain to lung cancer screening, the educator-trainee model could explore how the uptake of lung cancer screening is universally low across the United States (Haddad et al., 2020). Additionally, racial and ethnic differences in smoking patterns and age at diagnosis result in disparities in screening eligibility and therefore offering of an already underutilized EBP. Lung cancer incidence additionally varies in rural versus urban geographic areas, which may intersect with disparate environmental and occupational exposures (Haddad et al., 2020). Knowing these differences may help trainees address barriers to screening when identifying the appropriate patients to screen and advocating for patients who do not meet traditional guideline criteria but have significantly increased exposures and risks. Specific consideration to the issues of race, ethnicity, geography, and aspects of the local built environment surrounding the patient may be built into the clinical encounter. Educators could emphasize language and behaviors in the clinical encounter that promote trust and understanding between the trainee and patient to ensure the decision to screen or not is fully informed.

### Domain 2. Societal context

The societal context consists of three determinants (socio-political forces, economies and physical structures) that incorporate upstream, midstream and downstream social determinants that ultimately inform equitable EBP delivery. Educators could mention how economic structures (e.g., employment and insurance) may change access to necessary resources or suggest this is something the educator-trainee dyad might investigate further if unknown at the time of teaching. For example, differential coverage of medications or home medical equipment may work in concert with or against effective dissemination of an EBP (Emmons & Chambers, 2021). The educator could be explicit about how physical structures, such as where a hospital or clinic is situated within a city or town, may influence residents of that area as well as individuals who may travel to receive care there. Understanding crime and violence of a neighborhood, quality of housing, and accessibility of transportation may change if and how an EBP is recommended. With that context, trainees may be taught how additional resources may be sought so that historically and intentionally excluded groups of individuals reliably can access the EBP (Odeny, 2021). Recognition of sociopolitical forces includes understanding when law and social structure perpetuates oppression and discrimination (e.g., racism, classism, ableism, etc.). This is most relevant in public health policy related to an EBP, often in the context of education, preventive treatment, and screening.

When prescribing inhaler therapy for obstructive lung disease, the educator-trainee dyad might consider populations of patients who are frequently misdiagnosed with obstructive lung disease, why, and how this affects prescription of inhaler therapy (Pleasants, Riley, & Mannino, 2016). Specifically, the overlapping features of asthma and chronic obstructive pulmonary disease (COPD) may lead to incorrect diagnosis that is perpetuated over time and never re-explored. Access to specialty pulmonary services versus primary care may mediate this relationship (Sokol, Sharma, Lin, & Goldblum, 2015). Additionally, access to the single maintenance and rescue therapy inhalers based on pharmacy availability under different insurance plans is an example of how physical structure interacts with economy (Norris, Modi, & Al-Shaikhly, 2022). The trainee-patient interaction might focus on education including risk factors for developing different types of obstructive lung disease, exacerbating factors beyond smoke inhalation including indoor biomass fuel and inhalant toxins in the work setting (Kaji et al., 2014), and exploration of how clinicians may better assist patients in mitigating these exposures. Societal context related to obstructive lung disease relates to broader environmental and policy issues related to climate change (Joshi, Goraya, Joshi, & Bartter, 2020). Understanding how climate change has dynamically altered respiratory health over time is a relevant framework to consider how certain populations may be disparately affected. These upstream issues are often addressed by advocacy or research efforts.

### Domain 3. Culturally relevant factors of recipients

Educators, trainees, and patients each have unique characteristics and social identities based on their lived experiences. Trainees receive education on the delivery of EBPs to their patients and patients are the recipients of the EBPs directly. Reflecting on culturally-relevant factors as defined by the HEIF including one’s own implicit biases, socioeconomic status, race and ethnicity, health literacy, health beliefs, and trust of healthcare is important to examine language used in educator-trainee and trainee-patient communication. Implicit bias training has been integrated into medical education curricula readily (Gonzalez, Lypson, & Sukhera, 2021), as trainees are taught to consider how their experiences shape the way they communicate and interact with patients. Trainees additionally could intentionally learn demographics of their patient population to explore how living with these identities shapes one’s experience. Educators could provide this context and a safe space for discussing factors that contribute to health-related behaviors and motivations, during instructive sessions about EBPs. Understanding how patients identify themselves in society, and any intersecting identities they have, may lend to open conversations about barriers patients experience that may limit successful implementation of an EBP. Trainees could then use this engagement in culturally relevant factors of patients to garner trust and maintain intellectual curiosity.

In an example of tobacco treatment education, asking trainees to reflect on their lived experience with family members or friends who have suffered with tobacco addiction may provide insightful reflection on patients’ motivations for ongoing smoking, rather than framing the patient with a “habit” completely within their power. Alternatively, educators could share examples from their lives and practice if able or utilize a patient engagement program to allow a diverse group of patients to directly share their narratives with trainees (Rowland et al., 2019). Teaching how to respectfully query patients’ motivations to smoke, and explore connections between addiction and mental health disorders is a necessary piece of culturally relevant education. Additionally, shedding light on how systemic structures might enable tobacco use and certain individual health and social needs are related to tobacco use could be weaved into teaching history-taking and ways to establish rapport between clinicians and patients. (Leone et al., 2020)

### Proposed assessment

A 360-degree learner assessment could be utilized to assess the fidelity of the HEIF in medical education. Learners would provide a reflective self-assessment of relative knowledge in equitable care for each topic, before and after curricular interventions designed to teach about EBPs. This could be coupled with observer feedback (either by a peer or by the educator) on their implementation of the domains in context of a simulated case, a real-life scenario (possible in both inpatient and outpatient settings), or both in sequence. Soliciting patient or family member feedback could be sought as well, as the goal of utilizing the HEIF is to improve equitable patient-centered care. This results of this assessment might then be examined with regards to our three aforementioned domains to identify where and how barriers and facilitators to discussing each may present (i.e., the facilitation component of the framework). Exploration of gaps in discussion of each domain could focus on whether or not the HEIF context in teaching is suitable and sustainable for the EBP in question.

Future work that utilizes the HEIF in curriculum development might outline how each of the three domains is covered in the content of the curriculum. Using the specific considerations for exposure risk factors, disease risk factors, and complications of disease, clinicians will need to modify the content within each of these domains to adequately reflect what is known or being explored with regards to disparities for the EBP in question. Dissemination and implementation principles are evolving as a crucial element of the learning health system, and in this context would promote invested partner engagement from the community as well as both educators and learners (Trinkley, Ho, Glasgow, & Huebschmann, 2022). Barriers and gaps to implementation of the HEIF in curriculum development could be addressed systematically and simultaneously with a needs assessment, according to the levels of the framework: the clinical encounter, the recipients, the inner context, the outer context, or the societal context. When a barrier or gap is identified, it should be categorized to one or more level of the framework, and facilitation could occur to specifically identify if and how the barrier is overcome or gap is filled. For example, this may include a lack of faculty facilitators with expertise in equity concerns related to an EBP or a lack of time to provide this education to trainees in an ambulatory setting. Facilitators and content resources may be shared between programs (e.g., internal medicine, pediatrics, and family medicine), and faculty might devise brief teaching points (e.g., on a single aspect of preventive care) to discuss during every annual visit precepted with a trainee during a clinic session, as examples that address common barriers.

### Limitations

Although the HEIF provides a comprehensive process and framework for stakeholders and considerations in health equity, it is a research tool and therefore may not seamlessly translate to medical education. The HEIF is complex and involves consideration of multiple layers of individuals and systems to the delivery of an EBP. Although helpful in curriculum development, using the HEIF may not be possible nor appropriate for every EBP educators and learners encounter real-time during patient care experiences. A simplified version of the HEIF would likely better integrate in bedside teaching. As it is currently designed, the HEIF has a complex network of micro, meso, and macro levels of considering EBP implementation that are not discretely outlined. To be translated to bedside teaching, each component of the framework could be renamed to facilitate integration into clinical work rounds or the clinic room. For example, the circles surrounding the clinical encounter should reflect the parties involved in the EBP, most commonly the clinician and patient as well as caregivers, and possibly other staff who assist in delivering the EBP, and thus labeled the micro environment. In the meso environment, considerations to the patient’s day-to-day life may include the timing and execution of the EBP (i.e., scheduling the EBP, transportation to completing it, financial considerations for the patient). In the macro environment, social, economic, and political considerations of the EBP and how these intersect with the patient’s experience should be explored. This simplified framework could be utilized when the trainee is discussing the case either on rounds or in clinic with the educator, with a reference to considering one component from each environment. A background in implementation science and the theory behind the framework is helpful to understand its applicability and usability. For this reason, its use may be limited to academic clinical environments where expertise in implementation science is available.

The structure of the HEIF gives equal consideration to the different stakeholders involved in the clinical encounter. However, EBPs may require more of the recipient or clinician or system, depending on the innovation and intervention. The recipient is also framed as passive in the receipt of an EBP, but often play much more of a dynamic and integrated role in the operationalization of EBP delivery.

Furthermore, the HEIF does not directly consider the interaction between the recipient of the EBP and the health system. It places the recipient within the health system, but when actually attempting to obtain or complete the EBP, what are the barriers the recipient might experience? This is another aspect of care in which health equity and implicit bias should be considered, but is not confronted within the framework.

Lastly, the HEIF is founded upon a health disparities framework. Disparities are specifically differences in outcomes, including incidence or prevalence of disease and clinical outcomes such as morbidity and mortality. Equity-focused frameworks deal with the mechanisms of causes of health disparities. The HEIF could be restructured based on existing health equity theory, with specific attention to critical theories that identify long-standing structural causes of inequities (i.e., post-colonialism, structural violence, etc.) (Snell-Rood et al., 2021). Using a critical theory, the HEIF would be more adequately rooted in concepts beyond biomedical education, including sociology and anthropology. However, this may also change its applicability in a purely clinical context. Critical theories require deliberate reflection and critique of social norms in commonly held beliefs on a topic, and are used in research on various aspects of society and culture (Chow, Hirshfield, & Wyatt, 2022). This introspection forces acknowledgement of personal views and how those views shape experiences in a broader social and political context. Using a critical theory promotes analysis that can uncover new concepts and reframe connections between established themes. Critical Race Theory is an example critical theory that was used to understand how the legal system might be racially biased, and is now applied widely to understand the impact of racism in various fields, lending scrutiny to established power structures that work against social justice, and legitimizing the experience of minoritized individuals. In a related vein, critical postcolonial theory explores the narratives and lived experiences of minoritized groups, facing the history of colonization and long-standing impacts on modern culture and society. Considering these theories individually could provide new framing for the types of questions to consider within the HEIF, especially on the macro level querying sociopolitical forces that drive the delivery of EBPs to patients. This is particularly important because understanding macro level inequities may seem abstract in the context of patient care. For example, the deep roots of historical redlining are entrenched in colonialist social practices, and impact respiratory inequities experienced by Black American adults with asthma (Schuyler & Wenzel, 2022). Historical redlining itself is also a racist practice. Understanding how critical theory and postcolonial theory apply to this association of poorer outcomes for Black adults with asthma may be important to explicitly discuss the macro environment surrounding equity in inhaler therapy. Using critical theories also makes the recognition and unpacking of reasons for implicit bias systematic, which is relevant in considering the factors of individuals in the micro environment of the HEIF. Given the need for understanding the role of systemic racism and oppression in institutions of medical education, critical theories could illuminate unique equity considerations in the HEIF and delivery of EBPs (Chow et al., 2022).

## Conclusion

In looking forward to creating a physician workforce that values inclusivity and diversity, the HEIF provides a means to ensure evidence-based health care practices are equitably and fairly applied. With regards to current tools available to teach EBM, none explicitly incorporate health equity principles nor offer a structured format for identifying evidence-based practices that may be subject to implicit and explicit biases (Kumaravel et al., 2020). The HEIF therefore forces a population health level of thinking about an EBP, but specifically in the context a single clinical encounter.

Utilizing the HEIF to teach EBPs teaches trainees how to incorporate health equity principles in daily clinical care. The foundational domains of the clinical encounter, societal context, and culturally relevant factors of recipients are relevant in both the educator-trainee and trainee-patient dyads. The educator could focus on teaching the learner to delineate questions to ask surrounding the accessibility and feasibility of completing EBPs. The learner could in turn reflect on their own biases in asking these questions of their patients and consider innovative ways to deliver EBPs to ensure equitable care. The HEIF can be complex to dissect for those without a background in implementation science, and there is room to simplify its structure. Although health equity interventions stretch far beyond physician educator-trainee-patient relationships, a systematic discussion of how EBPs could be more fairly applied to all people is practical and broadly applicable. Teaching EBP delivery with the HEIF is one way to intentionally incorporate equity into medical education.

## Figures and Tables

**Table 1:** Developing or modifying innovations in education with an equity lens using the HEIF, with internal medicine-based examples

Health care delivery domain	Dyad	Determinant definition	Example in pulmonary medicine education of queries that may delineate determinants or moderators in implementation success or failure
Clinical encounter	Educator-Trainee	During healthcare appointments, how and why decisions regarding diagnoses and treatments are made	Lung cancer screening - Who is the target population for lung cancer screening? - How would you describe the process of lung cancer screening to a patient? - What are the expectations of our patient should screening come back positive or negative? - Which patients historically have lower rates of screening?
	Patient-Trainee		Lung cancer screening - What is the patient’s perception of lung cancer screening? - What is the patient’s interpretation of shared decision-making with lung cancer screening?
Societal context	Educator-Trainee	The role of historical or current economies, physical structures, and sociopolitical related to delivery of the EBP	Asthma or COPD inhaler therapy - Historically, which patient populations have the highest morbidity and mortality related to asthma or COPD? Why? - How much do inhalers cost? How is or is this not attenuated by insurance? - How has health policy shaped air quality in the preceding 50 years and what have been the effects on respiratory health? (Gaffney, Himmelstein, Christiani, & Woolhandler, 2021)
	Patient-Trainee		Asthma or COPD inhaler therapy - How does the patient contextualize the effect of their lung disease within their life? - Does the patient’s employment status or insurance status relate to their symptom severity? - Are there environmental exposures that relate to their symptom severity?
Culturally relevant factors of recipients	Educator-Trainee	Consideration of the lived experience of the individual to understand how implementation may need to be adapted	Tobacco treatment education - Is there a relevant lived experience personally, with a family member, or with a friend who has received tobacco treatment? - What is the perception of patients who smoke? - How does the health system view patients who smoke? Do you agree with this view? Why or why not? - What is the implication of terms such as smoking cessation versus tobacco treatment? Why is this difference important? - What populations might benefit the most from targeted tobacco treatment?
	Patient-Trainee		Tobacco treatment counseling - What are social and cultural influences on smoking? - What are barriers to tobacco treatment? - How does level of education influence ongoing smoking and tobacco treatment? (Nguyen-Grozavu et al., 2020)

COPD, chronic obstructive pulmonary disease; EBP, evidence-based practice

## Data Availability

Data sharing is not applicable to this article as no new data were created or analyzed in this study.
